# Electroacupuncture inhibited carrageenan-induced pain aversion by activating GABAergic neurons in the ACC

**DOI:** 10.1186/s13041-024-01144-z

**Published:** 2024-09-27

**Authors:** Yichen Zhu, Haiju Sun, Siqi Xiao, Zui Shen, Xixiao Zhu, Yifang Wang, Xiaofen He, Boyi Liu, Yongliang Jiang, Yi Liang, Janqiao Fang, Xiaomei Shao

**Affiliations:** Key Laboratory of Acupuncture and Neurology of Zhejiang Province, Department of Neurobiology and Acupuncture Research, The Third Clinical Medical College, Zhejiang Chinese Medical University, Hangzhou, 310053 China

**Keywords:** Pain aversion, Pain, GABAergic neurons, Anterior cingulate cortex, Electroacupuncture

## Abstract

Pain aversion is an avoidance response to painful stimuli. Previous research has indicated that the anterior cingulate cortex (ACC) is involved in pain aversion processing. However, as interneurons, the role of GABAergic neurons in the ACC (GABA^ACC^ neurons) in pain aversion is still unclear. Electroacupuncture (EA) has been shown to ameliorate pain aversion, but the mechanism is not clarified. The present study provided evidence that inhibition of GABA^ACC^ neurons contributed to pain aversion. EA alleviated pain aversion by activating GABA^ACC^ neurons in an intensity-dependent manner. Specifically, 0.3 mA EA stimulation showed better effects on pain aversion than 0.1 mA stimulation, which could be reversed by chemical genetic inhibition of GABA^ACC^ neurons. These results provide a novel mechanism by which EA alleviates pain aversion by reversing GABA^ACC^ neurons.

## Introduction

Chronic pain, as a global disease, reduces one’s quality of life [1], affects daily social activities, and places a significant economic burden on individuals and society [2, 3]. As one of the characteristics of pain [4], pain aversion is the awakening of pain sensation or prediction of nociceptive stimuli through pain memory to achieve conditional avoidance and is difficult to eliminate [5]. The reciprocal correlation of aversion and pain increases the difficulty of pain treatment and promotes the progression from acute pain to chronic pain [6–8]. Extinguishing/terminating aversive states can directly provide relief as well as reinforce behaviors that result in pain avoidance. However, the mechanism that alleviates pain aversion is poorly understood. To treat pain more effectively and prevent the progression of pain to chronic pain, exploring the mechanism of pain aversion is important for innovative therapy for chronic pain.

As a central hub for information processing throughout the brain, the ACC is activated in both acute and chronic pain [9]. The researchers have found that ACC involved in the integrating information [10] of pain related negative information, such as aversion [10], anxiety [11] and depression [12] and others. ACC plays a key role in coordinating the functions of other brain regions [13]. Our previous study focused on the relationship of ACC and pain induced negative emotion. For example, we have found that changes of synchronous neural oscillations in the ACC brain region in pain-aversion model [14], also found the electroacupuncture alleviated hyperalgesia and pain induced anxiety-like behaviors through activation of GABAergic neurons and GABA receptor in ACC [15]. The report indicated that damaging to the ACC region significantly affected pain aversion [16]. Above evidence suggested that the ACC may be critical for the formation of pain aversion. However, the specific mechanism by which the ACC regulates pain aversion still unclear.

GABA^ACC^ neurons transmit and preserve information in the brain [17] by affecting other neurons and transmitting transmitters [18, 19]. In addition, studies have found that severe pain stimulation can cause plasticity changes in GABAergic neurons [20] and change the release of neurotransmitters, such as GABA [21]. As GABA is an important neurotransmitter in the brain [22], changes in GABA content have a significant effect on pain, emotion, and cognition [23]. However, the specific mechanism of GABA^ACC^ neurons in regulating pain aversion still warrants further study.

Electroacupuncture (EA) treatment, as an important complementary and alternative therapy, has no obvious adverse reactions compared with opioid peptide drugs and other analgesic drugs [24, 25]. EA has multiple intervention effects, such as analgesic effects, emotional relief and cognitive improvement [26, 27]. To a certain extent, the intervention effects of EA are intensity dependent. A previous study showed that EA intervention could alleviate conditioned place aversion evoked by inflammatory pain [14]. The effects of EA intervention were considered to modulate the function of the ACC [28]. However, it is still unknown whether EA relieves pain aversion in an intensity-dependent manner by regulating the activity of GABA^ACC^ neurons.

Our previous experiments found that both pain formation and arousal stages can be accompanied by pain aversion. However, research has demonstrated that early intervention of pain aversion in the pain formation stage can prevent the establishment of pain-evoked plasticity in the brain similar to the memory mechanism, which is a potential breakthrough in the treatment of pain [29, 30]. Therefore, exploring the in-depth mechanisms of the ACC during the formation stage of pain aversion was the focus and direction of this research. In this study, we established mice with pain aversion by carrageenan injection into the hind paws. The role of GABA^ACC^ neurons in regulating pain aversion was identified by conditioned place aversion (CPA), fiber optic calcium imaging, chemical genetics, etc. The effect of different intensities of EA stimulation on mice with pain aversion was measured by immunohistochemistry. We concluded that 0.3 mA EA intervention alleviated pain aversion by increasing the activity of GABA^ACC^ neurons.

## Materials and methods

### Animals

All experiments used C57BL/6J adult male mice aged 8–10 weeks, which were provided by the Experimental Animal Centre of Zhejiang University of Chinese Medicine. It was important that the animals used in the experiments were certified by the Association for Assessment and Accreditation of Laboratory Animal Care. Before the experiment, the mice were fed adaptively for one week. During the feeding process, the mice were randomly divided into groups and raised in cages according to the grouping, with four to five mice in each cage. The bottom of the cage was covered with 3–5 cm thick corncob padding. The mice were provided with clean water and sufficient food in the cage. The environment was well ventilated, with alternating light and dark circulation for 12 h, and an air filtration system was provided.

### Animal models

Mice were respirationally anesthetized, and when they were fully anesthetized, they were fixed on a thermostatic table. After the sole of the left hind foot of each mouse was sterilized, 25 µl of 0.5% carrageenan (22049-5G-F, Sigma) was injected into the sole of its left hind foot. Then, mice were put back into the feeding room after they were awake. Successful modeling was marked by a decrease in the pain threshold of the left foot and CPA scores.

### Virus and trace injection

Mice were anesthetized by intraperitoneal injection of 0.3% pentobarbital and immobilized on a brain stereotyper (RWD, 68025, Shenzhen, China). Mice were kept warm with a heating pad (RWD, 69000, Shenzhen, China), and intracranial virus was injected using a microfuge with a volume of 80 µl per lateral nucleus (WPI, UMC4, Sarasota, FL, USA) at a rate of 60 nL·min − 1. To avoid virus spillage, the microfuge needed needs to be left in place for 8 min after injection. According to Paxinos and Franklin’s The Mouse Brain in Stereotaxic Coordinates (Fifth Edition), the location range of the ACC (AP, + 1.55 mm; ML, ± 0.35 mm; DV, -0.85 mm) was determined.

To investigate the effect of GABA^ACC^ neurons on pain aversion, we used chemogenetic techniques to specifically activate or inhibit neurons. We injected rAAV-VGAT1-hM3D(Gq)-mCherry-WPRE-pA (PT-489, Wuhan BrainVTA Scientific and Technical Corporation) or rAAV-VGAT1-mCherry-WPRE-pA (PT-0325, Wuhan BrainVTA Scientific and Technical Corporation) into the right ACC to activate GABA^ACC^ neurons. We injected rAAV-VGAT1-hM4D(Gi)-mCherry-WPRE-pA (PT-488, Wuhan BrainVTA Scientific and Technical Corporation) into the bilateral ACC for inhibition. Clozapine-N-oxide (CNO) (1 mg/mL) was injected (2 mg/kg, intraperitoneally) at 4 h, 1, 2 and 3 days. After the behavioral test, all mice were deeply anesthetized and perfused with 0.9% saline followed by 4% (w/v) paraformaldehyde. The brains were removed and stored in 4% paraformaldehyde at 4 °C for 24 h, and then they were dehydrated in different concentrations of sucrose solution (15% and 30%). Images of virus expression were obtained using a virtual slide microscope (VS120-S6-W; Olympus, Japan).

### Drug destruction of ACC

The ACC was destroyed by injection of quinolinic acid. The mice were then fixed on the stereotaxic frame. When we confirmed the position of the ACC, 150 nl quinolinic acid (15 mg/ml) or 150 nl saline was injected into the bilateral ACC in the corresponding group. Surgery was performed one week before carrageenan injection. All mice were allowed to recover for one week and were subjected to subsequent experiments.

### Fiber optic surgery

rAAV-VGAT1-Gcamp6m (PT-3317, acquired from Wuhan BrainVTA Scientific and Technical Corporation) was injected into the right ACC brain region, and a ceramic needle (R-FOC-BL200C-39NA, Shenzhen, China) was slowly inserted 0.1 mm from the viral injection site. The ceramic pins were then fixed to the skull surface with glue. Two sterile flat-head pins were then transferred into the skull for optical fiber fixation. To avoid the influence of external light on the experiment, black dental cement mixed resin was used to cover the calvarium, optical fiber, and exposed skull. Carrageenan injection was performed three weeks after surgery.

### Fiber optic calcium imaging

Before the experiments started, MATLAB 2017b software was run, and the fiber ends were placed in a dark environment by adjusting the desired parameters. After the calcium signal was stabilized, for each group of mice, fiber optics and a tricolor fiber optic signal recorder fiber optic instrument (QAXK-FPS-TC-LED-FM, Shenzhen, China) were connected to a ceramic cannula that had been placed in the brain of each mouse to record the fluorescence signal during all aspects of behavior. The fiber optic recording system (405 nm and 470 nm) was applied to acquire neuronal Ca^2+^ transients in mice in different states. Fluorescence intensity was analyzed by calculating (F-F0)/F0, where F0 is the baseline fluorescence signal and F is the signal of Ca2 + change in the target neuron. Changes in fluorescence intensity were analyzed by examining the change in fluorescence intensity from 2 s before to 10 s after the injurious stimulus and 6 s before and after mice entered the pain box.

Paw withdrawal thresholds (PWTs) were incorporated to observe the changes in ACC neuron activity when mice were subjected to suprathreshold mechanical stimulation in physiological and pain states; CPA was incorporated to observe the changes in ACC neuron activity during the baseline phase and test phase when each group of mice entered the pain paired chamber.

### Behavioral tests

#### Von frey filament test (PWTs)

PWTs were measured using the classical von Frey method. The mice were placed on a metal net, covered with an opaque hood, and acclimatized for 30 min. Then, we stimulated the hindpaws of the mice with von Frey fibers in turn from small to large. A positive response was recorded if the mice showed rapid retraction or licking of the foot after stimulation. PWTs were measured at baseline and at 4 h, 1 day, 3 days, and 5 days.

### Conditioned place aversion (CPA)

CPA was used to examine pain aversion behaviors in mice. The two chambers have dividable doors for the mice to pass freely. The walls of the two chambers had clearly distinguishable visual cues, one with black horizontal stripes on a white background and the other with black vertical stripes on a white background. The mice were acclimatized in the room 30 min before the experiment, with the wall lights on and with the room at 24 °C. To ensure the effectiveness of the experiment each time before putting the mice into the box, the box was disinfected with 75% alcohol and then wiped with water, to avoid the smell residue, using kitchen paper to wipe excess moisture so that it would be dry and odorless before the start of the experiment. The test was performed in three consecutive phases: the preconditioning stage, conditioning stage, and postconditioning stage.

In the first stage (preconditioning stage), the partition door was opened, the mice entered the experimental chamber from one side of the chamber in each half, and the mice moved freely in the two chambers for 10 min. Mice that spent more than 80% of the total time in one chamber were excluded. The second stage (conditioning stage) was divided into two parts, and the doors were closed during the entire period. Mice were randomly placed in a chamber (nonpain paired chamber) for 30 min the day before the injection of carrageenan, and the experiment was performed once. The next day, carrageenan was injected (day 0). Four hours later, mice were placed in the box contralateral to the previous day (pain paired chamber) for 30 min, and the left hind foot was stimulated with 1 g von Frey filaments from the 10th to the 20th minute at a frequency of 3–5 times/min; this process was repeated daily for 3 days. In the third stage (postconditioning stage), the partition door was opened, and the mice were allowed to move freely in both boxes for 10 min. The duration each mouse stayed in the two boxes was recorded separately, performed 1 time, at 3 days after one injection of carrageenan. The residence time in the chambers were compared before and after conditioning.

CPA Score = time in conditioned box after conditioning - time in conditioned box before conditioning.

### EA

Acupuncture points equivalent to “ST36 (Zusanli)” and “SP6 (Sanyinjiao)” were selected on both hind limbs. The acupuncture needle size was 0.16 × 7 mm, and the penetration depth was approximately 5 mm. We used a HANS acupoint nerve stimulator (HANS-200 A Huawei Co., Ltd., Beijing, China). The stimulation parameters were as follows: frequency 2/100 Hz; intensity 0.1 mA or 0.3 mA; and 30 min per treatment, once a day. The intervention times were 4 h and 1, 2 and 3 days after the first injection of carrageenan. It was necessary to observe whether there was any muscle twitching after stimulation. If there was no change, we adjusted the angle and depth of needle feeding. The remaining mice were given the same treatment but they did not receive stimulation. The whole operation was performed when the mice were awake. The Carr + hM4D + EA group and Carr + mCherry + EA group received EA treatment half an hour after the intraperitoneal injection of CNO.

### Immunohistochemistry

Mice were anesthetized by intraperitoneal injection of sodium pentobarbital. The thoracic cavity was opened under complete anesthesia, and 0.9% saline prechilled at 4 °C in the refrigerator was instilled into the heart cavity of the mice to replace the blood. After the eyes and liver of the mice were whitened, the whole brain was removed quickly after switching to slow perfusion with 4% paraformaldehyde. Fresh tissue was placed in a 4 °C refrigerator and immersed in 4% paraformaldehyde solution overnight, followed by sequential dehydration using 15% (w/v) and 30% (w/v) sucrose solutions until the tissue sank. Coronal Sect. (20 μm) were cut using a cryostat frozen sectioner (Thermo Fisher Scientific, NX50, USA). The brain slices were heated in a 37 °C water bath for 1 h and then washed 6 times with TBST for 10 min each time, and the speed was generally controlled at 80–90 rpm. Subsequently, the brain slices were blocked with 10% goat serum (S9100, Hangzhou, China) and 0.3% Triton X-100 (T8200, Solarbio, Beijing, China) closure solution and placed in a 37 °C water bath for 1 h. The brain slices were then incubated overnight at 4 °C with primary antibodies, including rabbit anti-c-Fos (1:500, ab190289, Abcam, USA) and rabbit anti-GABA (1:200, GTX125988-S, GeneTex, USA). The sections were then rewarmed for 1 h at 37 °C, again washed six times for 10 min each using TBST, and finally incubated with donkey anti-rabbit 488 (1:1000, A21208, Thermo Fisher, USA) in a 37 °C water bath for 1 h. After six TBST washes, the brain slices were coated with 4,6-diamidino-2-phenylindole (DAPI ab104139, Abcam, USA) to seal the slices. Images of virus expression were obtained using a virtual slide microscope (VS120-S6-W; Olympus, Japan).

### Statistical analysis

All experimental data are expressed as the means ± standard errors of the means (SEMs). Two-way repeated-measures analysis of variance (rmANOVA), followed by Tukey’s post hoc test, was used to statistically analyze the results of the PWT assessment. One-way analysis of variance (ANOVA) followed by the LSD post hoc test was used to statistically analyze the results of the CPA. Independent-sample t tests were performed to evaluate c-Fos expression. *P* < 0.05 was used as the criterion for a statistically significant difference.

## Results

### Pain aversion was induced by the injection of carrageenan

**Fig. 1:**
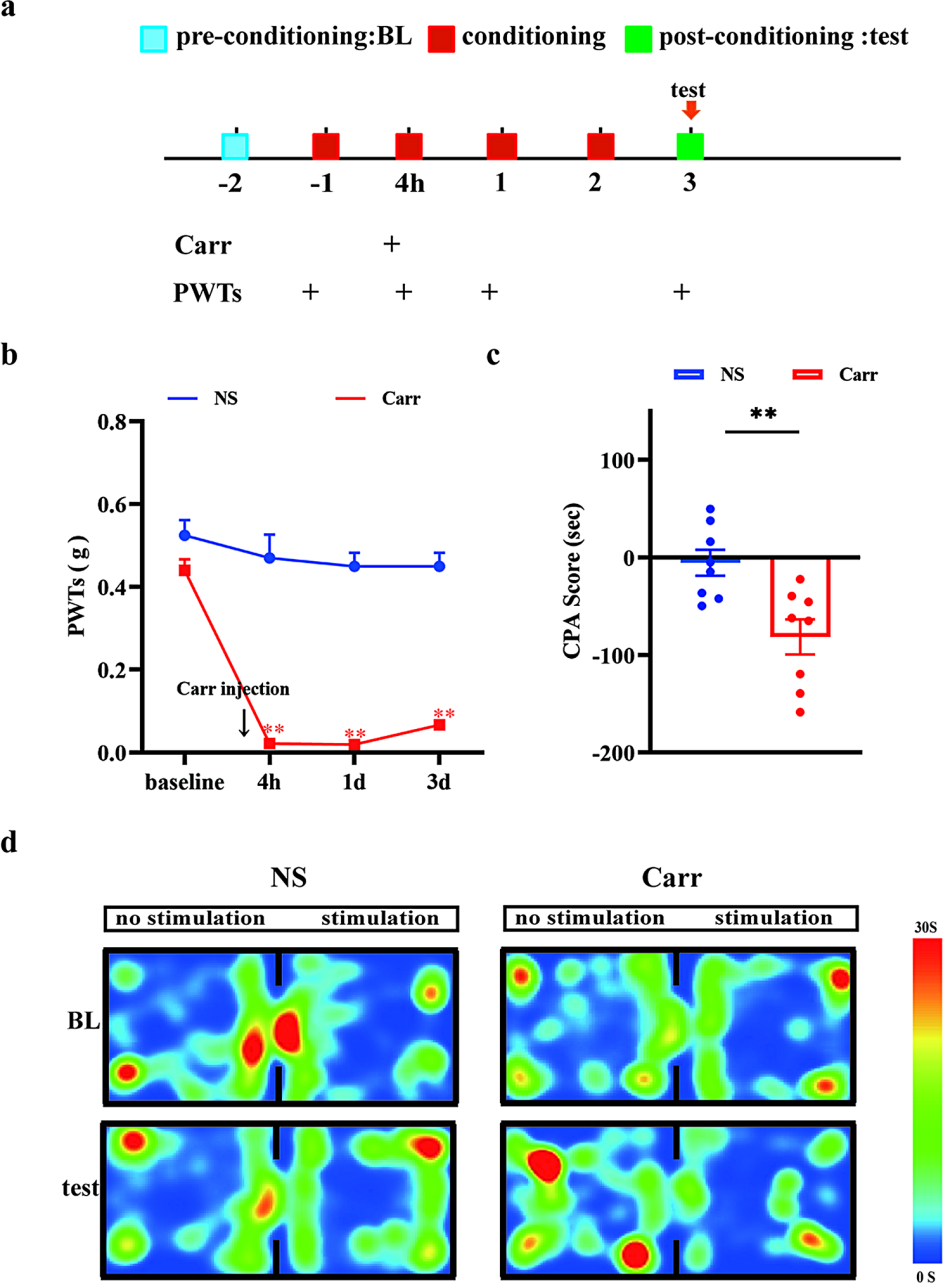
Injection of carrageenan induced pain aversion **(A)** Flow chart for this part of the experiment. **(B)** Comparison of the left-paw withdrawal thresholds of the NS group and Carr group at different times. **P < 0.01 compared with the NS group. **(C)** CPA scores of mice in the NS group and Carr gro**(D)** up. Representative graphs of CPA thermograms for each group

To explore the aversion induced by pain sensation, we established mice with pain aversion by carrageenan (Carr, 0.5%, 25 µL) injections. The mechanical paw withdrawal thresholds were tested by von Frey tests, and aversive behaviors were detected by CPA tests. The detailed time schedule of this part is depicted in Fig. 1A. At first, mice were placed in the CPA maze (two chambers with a doorway), and mice could freely explore the chamber; the time the mice spent exploring the two chambers was recorded separately. Then, carrageenan was injected into the left hind paws of the mice. Four hours after the first injection, the mechanical paw withdrawal thresholds of Carr-injected mice decreased significantly (*P* < 0.0001). Then, the mice were kept in one of the chambers randomly (stimulation chamber, 30 min per day, 3 consecutive days). Four days after the first injection, mice were placed in the maze and allowed to explore again. As shown in the figure, mice injected with Carr spent less time in the stimulation chamber than mice injected with normal saline (*P* = 0.0042), indicating that the model with pain aversion was successfully established (Fig. 1C and D). Thus, it was suggested that injection of carrageenan can induce pain aversion.

### ACC was involved in the processing of pain aversion

**Fig. 2:**
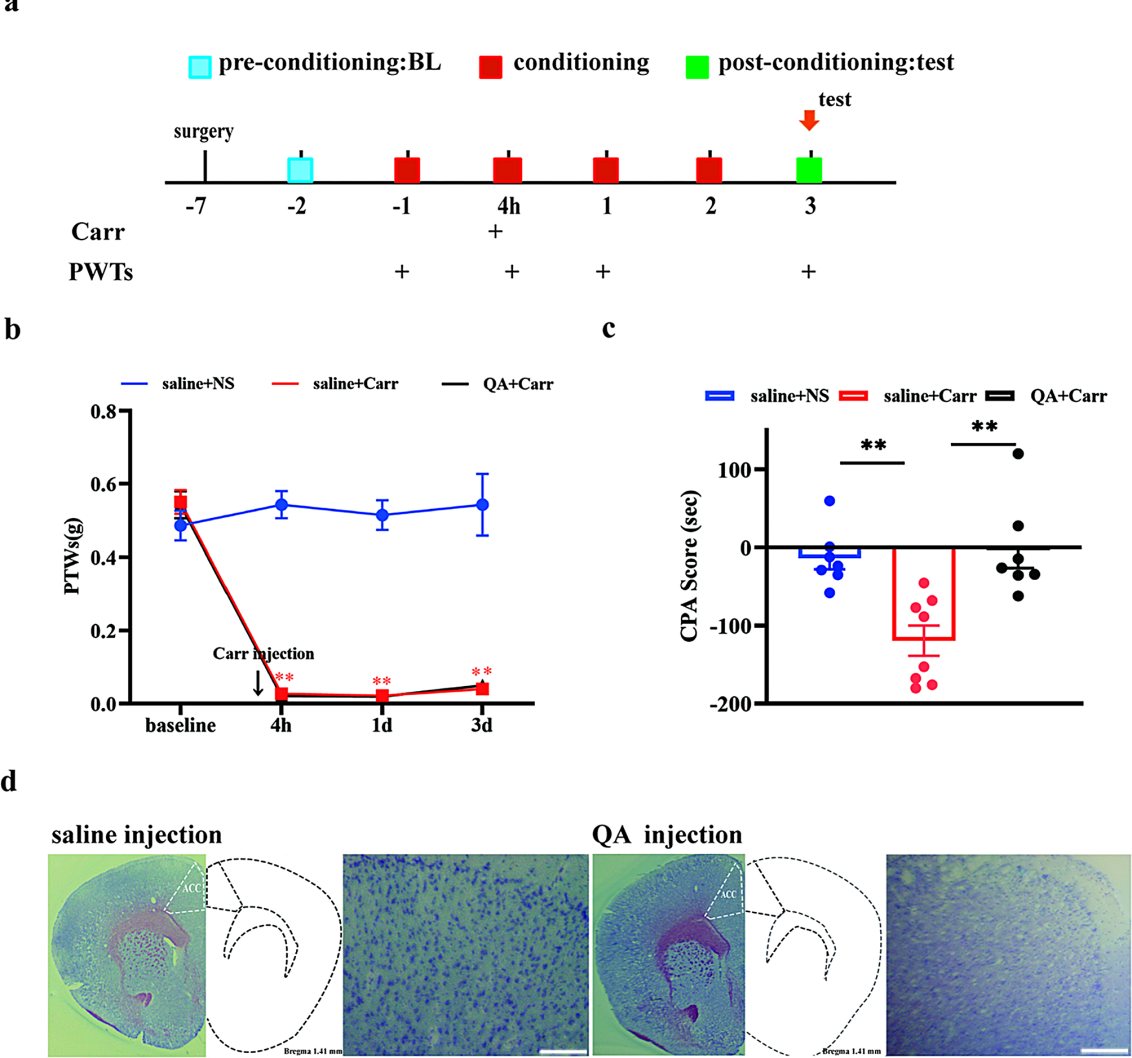
The ACC is involved in the formation of pain aversion. **(A)** Flow chart of the experiment. **(B)** Mechanical pain threshold of the saline + NS, saline + Carr and QA + Carr groups. Compared with the saline + NS group, **P < 0.01; compared with the saline + Carr group, ^##^*P* < 0.01. **(C)** CPA scores of the saline + NS, saline + Carr and QA + Carr groups. Pairwise comparisons **P < 0.01. **(D)** Schematic diagram of drug destruction in ACC brain regions, scale bar, 500 μm

To verify whether the ACC was involved in pain aversion, we injected quinolinic acid (QA) into the bilateral ACC to destroy the neurons at -7 days. The mechanical paw withdrawal thresholds were assayed at t -1 day, 4 h, 1 day and 2 days after carrageenan injection (Fig. 2A). CPA tests were performed as described previously. There was a significant decrease (*P* < 0.0001) in the mechanical pain threshold and the CPA score was higher (*P* = 0.0026) in the saline + Carr group than in the saline + NS group. This result suggested that severe pain and aversive pain were produced. Notably, the mice in the QA + Carr group showed no significant aversive behavior (compared with the saline + Carr group, *P* = 0.0011), while the mechanical paw withdrawal thresholds had no significant difference compared with the saline + Carr group (*P* = 0.9872). These results suggested that damage to the area of the ACC could inhibit the aversion behaviors induced by carrageenan but had no effects on the nociception induced by Carr (Fig. 2B and C). The above results suggest that the ACC was involved in aversive pain information processing, but it did not affect pain sensation. This result indicated that there were distinct mechanisms responsible for the formation of pain sensation and pain aversion, manifesting a phenomenon of separation.

### Activity of GABA^ACC^ neurons decreased in mice with pain aversion

**Fig. 3:**
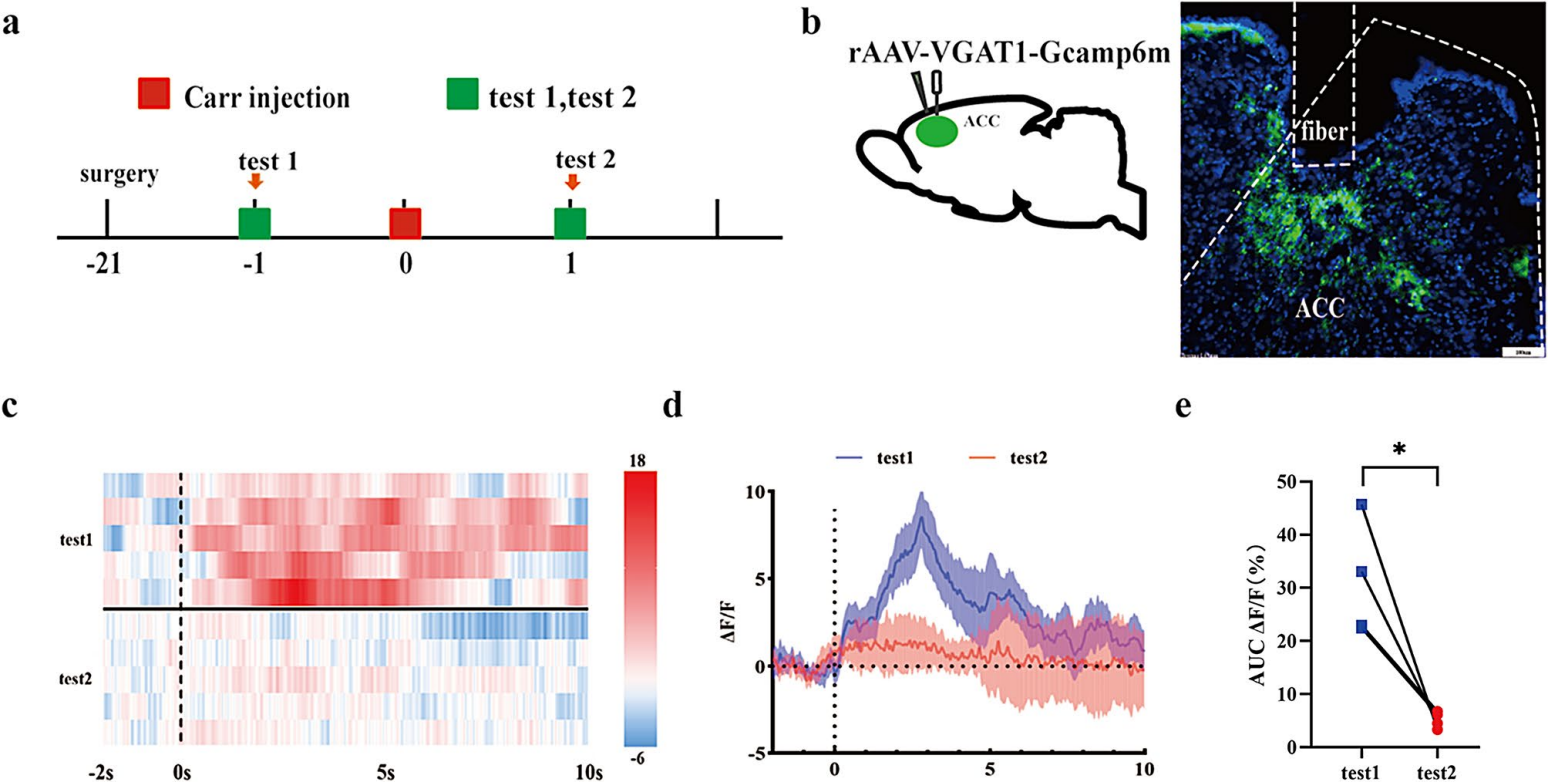
Decreased activity of GABA^ACC^ neurons in carrageenan-induced inflammatory pain model mice induced by mechanical pain stimulation. **(A)** Flow chart of the experiment. **(B)** Sagittal map and location diagram of virus injection and optical fiber implantation, scale bar, 100 μm. **(C)** Representative ΔF/F heat chart (from one mouse, 5 trials) of activity changes in ACC GABAergic neurons before and after Carr injection. **(D)** Representative ΔF/F line chart (from one mouse, 5 trials) of activity changes in ACC GABAergic neurons before and after Carr injection. **(E)** AUC diagram (0–10 s) of the activity of GABAACC neurons before and after Carr injection, pairwise comparisons, *P < 0.05. (*n* = 4 mice)

To further investigate the in-depth mechanisms of the ACC in pain aversion, we investigated the changes in the activity of GABA^ACC^ neurons during both pain stimulation and pain aversion by optical fiber calcium imaging combined with behavior tests. On day − 21, rAAV-VGAT1-Gcamp6m was injected into the right ACC, and optical fibers were implanted 0.1 mm above the injection site of the virus to record the activity changes in GABAergic neurons. After the virus was fully expressed, the activity changes in GABAergic neurons in the ACC brain region under pain and pain aversion conditions were detected (Fig. 3B). The activity of GABA^ACC^ neurons was significantly decreased after carrageenan injection (*P* = 0.0001) compared to the physiological state on day − 1 (Fig. 3C-E).

**Fig. 4:**
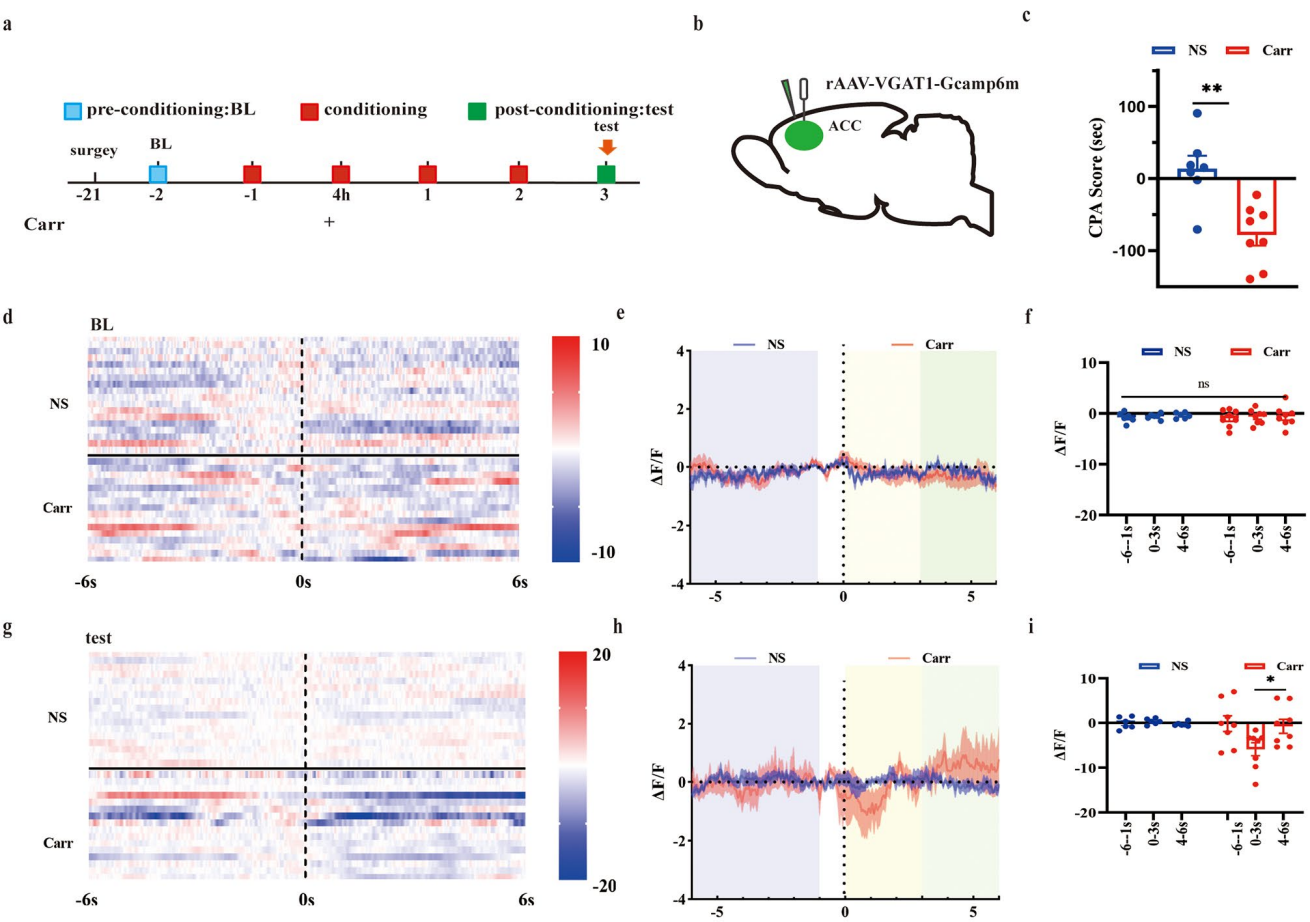
Changes in the activity of ACC GABAergic neurons during the formation of pain aversion. **(A)** Flow chart of the experiment. **(B)** Sagittal map of virus and optical fiber implantation. **(C)** CPA scores of mice in the experiment. Compared with the NS group, **P < 0.01. **(D)** Thermography of GABAergic neurons in the NS group and Carr group before and after entering the pain box at baseline. **(E-F)** ΔF/F line graph and area under the curve of ΔF/F at the baseline state. **(G)** Thermography of GABAergic neurons in the NS group and Carr group before and after entering the pain box under test conditions. **(H-I)** ΔF/F line graph and area under the curve of ΔF/F in the test state, pairwise comparisons, *P < 0.05

To detect the activity of GABA neurons in mice with pain aversion, we observed and recorded the activity of GABAergic neurons in the behaviors of pain aversion by optic fiber recording (Fig. 4A). We recorded the neuronal activity of all the mice before carrageenan injection (-2 days). The NS group and Carr group mice showed no significant difference in the activity of GABAergic neurons in any period (Fig. 4D-F). On day 3, while mice moved from the no-stimulation chamber (-6 s to -1 s) to the stimulation chamber (0 s to 6 s), activity of GABAergic neurons in Carr group showed a significant decrease in the 1–3 s period (*P* = 0.0377). However, in the NS group, there was no difference in any period (Fig. 4G-I). These results suggested that the activity of GABA^ACC^ neurons was inhibited under the pain aversion condition.

### The formation of pain aversion was blocked by activation of GABA^ACC^ neurons

**Fig. 5:**
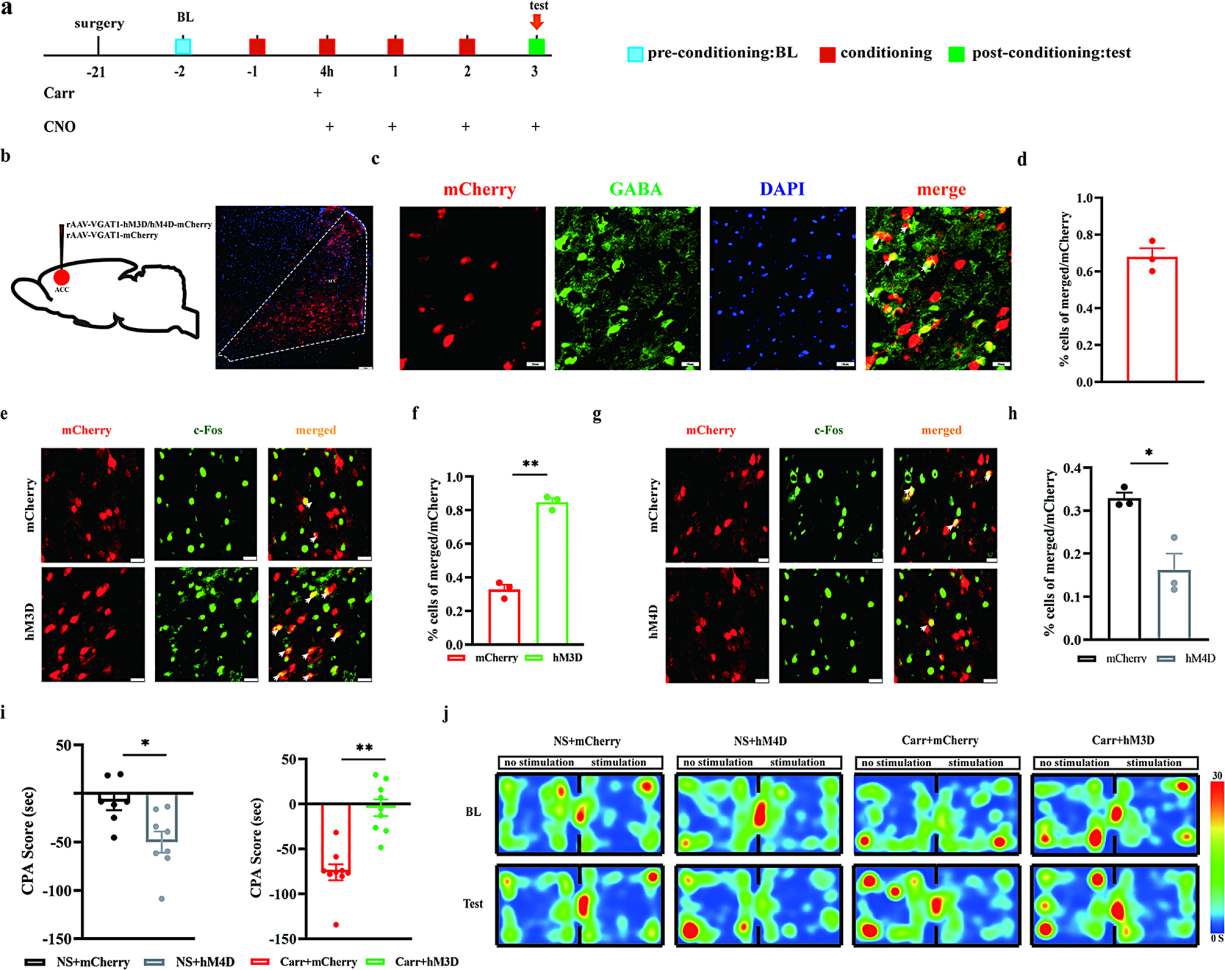
Effect of chemical genetic modulation of GABA^ACC^ neurons on pain aversion. **(A)** Flow chart of the experiment. **(B)** Schematic of chemog**(C)** enetic virus injection (left), schematic of mCherry-labeled cells (right) scale bar, 100 μm. Colocalization of mCherry (red) and GABA in the ACC brain region, scale bar, 20 μm. **(D)** Percentage of mCherry and GABA colocalization (*n* = 15, 3 mice) GABA green **(E)** and **(G)** ACC Representative maps of GABAergic neurons colabeled with c-Fos, c-Fos green, GABAergic neurons red. Scale bars, 20 μm. **(F)** and **(H)** Percentage of ACC GABAergic neurons colabeled with c-Fos (*n* = 15, 3 mice). Pairwise comparisons, **P < 0.01. **(I)** Experimental results of CPA in the NS + mCherry group, NS + hM4D group, Carr + mCherry group and Carr + hM3D group, pairwise comparisons, **P < 0.01. **(J)** Representative graphs of CPA th**(J)** ermograms for each group

As the mechanical pain threshold in mice remained unaffected by the destruction of the ACC, in the following experiments, we focused on the mechanisms of GABA^ACC^ neurons involved in the behaviors of pain aversion. Previous studies have shown that the activity of GABA^ACC^ neurons is reduced in a state of pain aversion. We explored the intervention that modulates GABA^ACC^ neurons on the behaviors of pain aversion by a chemical genetic test combined with CPA tests. Prior to the injection of carrageenan (-21 days), rAAV-VGAT1-mCherry or rAAV-VGAT1-hM3D-mCherry was injected into the right ACC brain region to activate GABA^ACC^ neurons; rAAV-VGAT1-mCherry or rAAV-VGAT1-hM4D-mCherry was injected into bilateral ACC brain regions to depress GABA^ACC^ neurons. Three weeks after virus injection, corresponding viral expression was observed in the ACC brain region (Fig. 5B). Virus specificity was verified by mCherry colocalization with GABA, and we observed that most mCherry-labeled VGAT1 was immunoreactive to GABA (Fig. 5C-D). The activation of the virus was clarified by the percentage of c-Fos-labeled neurons colocalized with mCherry. Compared with the control virus, activation of GABAergic neurons was significant in the hM3D group of mice (*P* < 0.0001) (Fig. 5E-F), and suppression of GABAergic neuronal activity was significant in the hM4D group of mice (*P* = 0.0117) (Fig. 5G-H). CPA was performed half an hour after intraperitoneal injection of CNO and 4 h, 1, 2 and 3 days after carrageenan injection. CPA scores increased in the NS + hM4D group compared with those in the NS + mCherry group (*P* = 0.0128) and decreased significantly in the Carr + hM3D group compared with those in the Carr + mCherry group (*P* < 0.0001) (Fig. 5I). The above findings suggested that inhibition of GABA^ACC^ neurons in the physiological state could induce aversive behaviors. Activation of GABA^ACC^ neurons in the pain state can block the formation of pain aversion.

### EA (0.3 mA) effectively blocked pain aversion behaviors

**Fig. 6:**
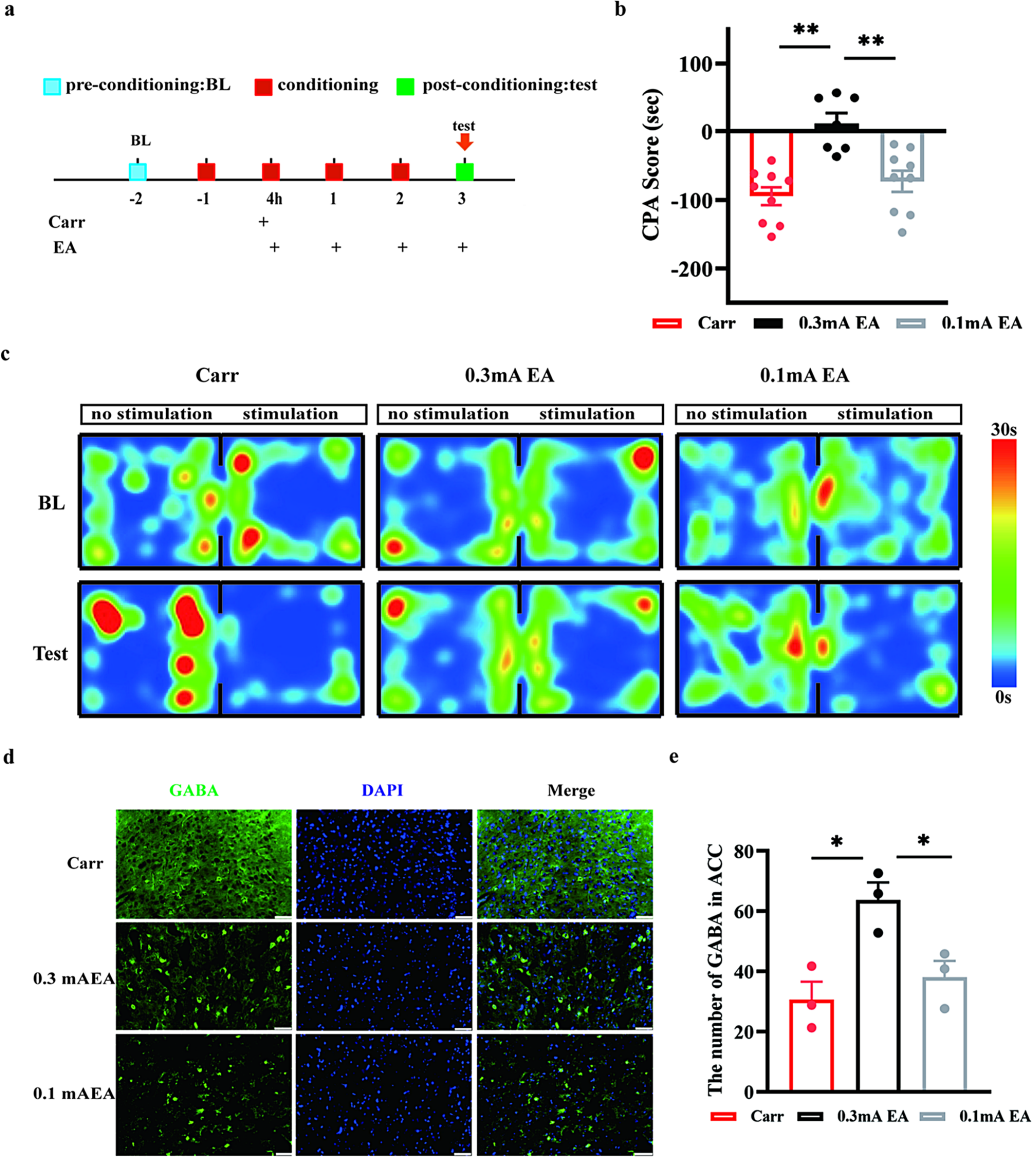
The effect of different intensities of EA on pain aversion behaviors. **(A)** Flow chart of the experiment. **(B)** Results of conditioned CPA in each group, pairwise comparisons, **P < 0.01. **(C)** Representative heatmap of conditioned aversion experiments in the Carr group, 0.3 mA EA group, and 0.1 mA EA group. **(D)** Colocalization of GABA (green) with DAPI (blue) in the ACC brain region in each group (*n* = 15, 3 mice). Scale bar, 50 μm. **(E)** Statistical map of the number of GABA neurons in the ACC in each group, pairwise comparisons, **P < 0.01

EA has a therapeutic effect on pain aversion to a certain extent, but it is still not clear whether different intensities of EA have different effects on blocking the behaviors of pain aversion. Thus, we measured 2/100 Hz EA interventions at 4 h, 1, 2 and 3 days after carrageenan injection. Two EA intensities of 0.1 mA and 0.3 mA were screened by observing the changes in CPA scores after conditioning. Compared with those of the Carr group, the CPA scores did not significantly change in the 0.1 mA EA group (*P* = 0.5365); moreover, the CPA scores decreased significantly in the 0.3 mA EA group (*P* = 0.0018) and were significantly different from those in the 0.1 mA group (*P* = 0.0002) (Fig. 6B). Histological validation of the Carr, 0.1 mA EA and 0.3 mA EA groups revealed that ACC GABA expression was different among the groups. GABA expression in the 0.3 mA EA group was significantly higher than that in both the Carr group (*P* = 0.0153) and the 0.1 mA group (*P* = 0.0444). Although GABA expression in the 0.1 mA EA group showed an upward trend, there was no significant difference (*P* = 0.6506) (Fig. 6E). These results suggested that at 2/100 Hz, 0.3 mA EA effectively blocked pain aversion. This result might be related to downregulation of GABAergic neuronal activity within the ACC.

### EA (0.3 mA) blocks pain aversion by modulating GABA^ACC^ neuronal activity

**Fig. 7:**
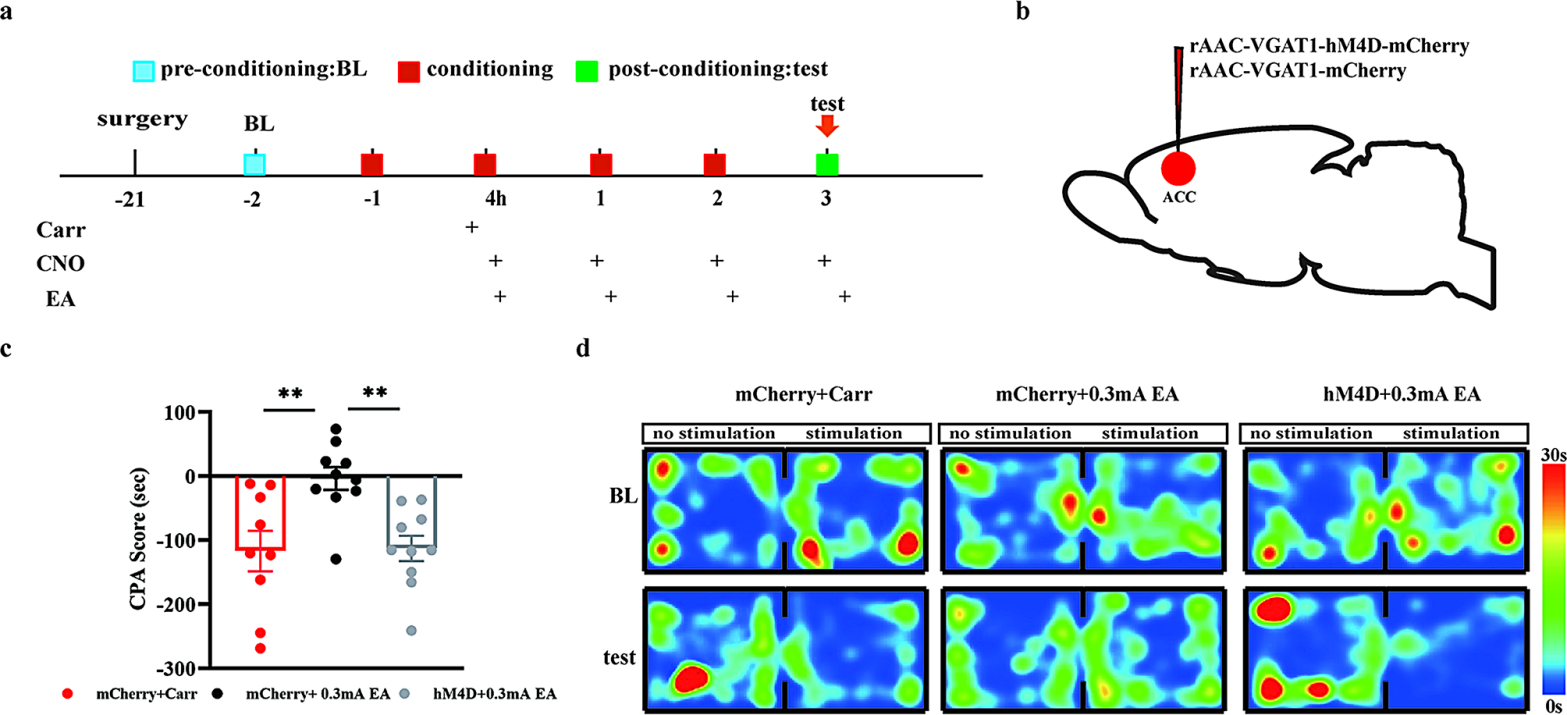
EA regulates pain aversion behaviors through GABA^ACC^ neurons. **(A)** Flow chart of the experiment. **(B)** Schematic diagram of viral injection sites. **(C)** CPA results of mice in the mCherry + Carr group, mCherry + 0.3 mA EA group, and hM4D + 0.3 mA EA group; pairwise comparisons, **P < 0.01. **(D)** Representative heatmap of the conditioned aversion experiment for each group

Next, to verify whether the preventive effects of 0.3 mA EA on pain aversion behaviors were associated with GABA^ACC^ neurons, we inhibited GABA^ACC^ neuronal activity by injecting rAAV-VGAT1-mCherry or rAAV-VGAT1-hM4D-mCherry virus bilaterally into the ACC before carrageenan injection (-21 days). EA was performed half an hour after intraperitoneal CNO injection (4 h, 1, 2, and 3 days after carrageenan injection) (Fig. 7B). CPA scores were significantly decreased in the mCherry + 0.3 mA EA group compared to those in the Carr group (*P* = 0.0056) and significantly increased in the hM4D + 0.3 mA EA group compared to those in the mCherry + 0.3 mA EA group (*P* = 0.0061) (Fig. 7C).

The above results suggested that 0.3 mA EA played a blocking role in pain aversion by activating GABA^ACC^ neurons.

## Discussion

The present study mainly found that pain aversion induced by acute inflammatory pain was closely related to the activity of GABA^ACC^ neurons. Inhibiting GABA^ACC^ neurons during pain aversion could effectively block pain aversion. EA relieved pain aversion in an intensity-dependent manner by improving the activity of GABA^ACC^ neurons.

A previous pain study focused on pain sensation and the corresponding behavioral response to injurious stimuli [31, 32]. We explored temporal changes by analyzing mice in the pain environment to determine whether pain aversion was produced. The environment of pain aversion was formed by combining the pain sensation induced by plantar carrageenan injection with a specific environment. On this basis, the formation mechanism of pain aversion was discussed.

The ACC is the relay station of pain-related information processing [33], which can convert pain sensation into pain consciousness [34], and the ACC plays an important role in the behaviors of pain [35]. Moreover, the ACC could predict pain and assess stimulus signals to determine whether to adopt an avoidance response by distinguishing or inducing nociception [36], which might be closely related to the formation of pain aversion and the consolidation of pain-related memories [31]. Furthermore, subjective pain perception may be exacerbated when the ACC overpredicts pain stimulus signals [37]. In this study, bilateral lesions of the ACC before carrageenan injection could block the formation of pain aversion, which verifies the involvement of the ACC in pain aversion. However, bilateral lesions of the ACC did not interfere with pain formation. It was verified that the ACC plays an important role in pain aversion.

Altered synaptic plasticity of neurons within the ACC was proven to be closely related to pain development [38] and pain-related negative emotions [39]. Studies have found that altered synaptic plasticity cannot be separated from abnormal neuronal excitation in the ACC [40, 41]. Abnormal neuronal excitation promotes the expression of pain aversion [42]. GABAergic neurons are important inhibitory neurons in the nervous system. GABAergic neurons can modulate the output balance of the ACC by affecting the local Ga2 + concentration, modulate neuronal excitability [43] and reduce the release of excitatory transmitters [44, 54]. Thus, we hypothesized that GABA^ACC^ neurons were important in pain aversion. In the present study, the activity of GABA^ACC^ neurons significantly decreased in mice with pain aversion behaviors. The activation of GABAergic neurons by chemical genetic specificity could block the development of pain aversion. Conversely, inhibition of GABA^ACC^ neurons in mice in a physiological state could induce conditional place aversion, which is similar to pain aversion.

In terms of analgesic methods, medications commonly used in the treatment of analgesic drugs have adverse effects, such as addiction and gastrointestinal reactions [45, 46]. EA, as a complementary and alternative therapy, is widely used to reduce pain intensity [47], shorten the duration of pain [48] and alleviate pain-related aversion [49]. EA has been proven to upregulate the NMDA signaling pathways of the high central nervous system [50], activate the activity of GABAergic neurons [51], and block synaptic loss and changes in the synaptic structure of neurons [52]. In addition, the selection of acupuncture points and the variation in electroacupuncture intensity have different effects on the disease [53]. Our previous study confirmed that EA could alleviate the intensity of pain and related negative emotions [54, 55] and prevent the recurrence of pain memory [56] by stimulating the bilateral ST36 and SP6. Thus, we applied 0.1 mA and 0.3 mA EA stimulation to this group of acupoints. Only 0.3 mA EA stimulation effectively blocked pain aversion. The expression of GABA transmitters was increased in the ACC of mice stimulated by 0.3 mA EA. Considering that GABA transmitters are released by GABAergic neurons, we suspected that EA alleviated pain aversion by activating GABA^ACC^ neurons. Thus, we inhibited GABA^ACC^ neurons by chemical genetics before EA intervention, which could block the effect of 0.3 mA EA. This proved that 0.3 mA EA played an anti-pain aversion role by activating GABA^ACC^ neurons.

Overall, these results suggested that the anti-pain aversion effects of EA on inflammatory pain model mice may be mediated by activation of GABA^ACC^ neurons. In addition, EA required a certain intensity to alleviate the inhibitory state of GABA^ACC^ neurons, which was complementary to the understanding of pain aversion and the mechanism of acupuncture treatment. The limitations of this study were as follows: Although 0.1 mA and 0.3 mA stimulation were found to produce different effects, the mechanism underlying this difference was not explored in depth. The mechanism of pain aversion has mainly been explored, but the feedback of pain aversion to pain and the mechanism of aversive arousal have not been studied.

## Data Availability

The original contributions presented in the study are included in the article/supplementary materials, further inquiries can be directed to the corresponding authors.
